# Insights into the Structural and Nutritional Variations in Soluble Dietary Fibers in Fruits and Vegetables Influenced by Food Processing Techniques

**DOI:** 10.3390/foods14111861

**Published:** 2025-05-23

**Authors:** Wenjie Sui, Shuiqing Wang, Yue Chen, Xiaoxuan Li, Xin Zhuang, Xinhuan Yan, Ye Song

**Affiliations:** 1 Jinan Fruit Research Institute, All-China Federation of Supply & Marketing Co-Operatives, Jinan 250014, China; yanjuan1206@126.com; 2 State Key Laboratory of Food Nutrition and Safety, College of Food Science and Engineering, Tianjin University of Science & Technology, Tianjin 300457, China; 22844949@mail.tust.edu.cn (S.W.); 22844948@mail.tust.edu.cn (Y.C.); 23842063@mail.tust.edu.cn (X.L.); 22842074@mail.tust.edu.cn (X.Z.)

**Keywords:** soluble dietary fiber, food processing technology, structural properties, nutritional properties, fruits and vegetables

## Abstract

Fruits and vegetables represent important dietary sources of soluble dietary fiber (SDF), a functionally essential component that contributes substantially to human health maintenance. The molecular structure of SDFs in fruits and vegetables is influenced by food processing techniques, which can contribute to improving the physiological activities of SDFs and promoting health benefits. This article presents a systematic review of the effects of common processing methods mainly involving drying, heating, powdering, fermentation, etc., on the structural and nutritional properties of SDFs, particularly focused on structural changes in molecular weight, monosaccharide composition, and functional groups, as well as nutritional functions including obesity prevention, hypolipidemic and hypoglycemic properties, etc. Processing-induced structure variations in SDFs inevitably change their fermentability and gelling ability, promote the growth of beneficial bacteria and the production of short-chain fatty acids, enhance immunity, and reduce the risk of chronic diseases. This highlights the prebiotic efficacy and metabolic disease intervention potential of processing methods to moderate SDFs by altering their structure. This paper comparatively summarizes the effects of physical, physicochemical, and biological processing technologies on the common structural and nutritional properties of SDFs, aiming to provide theoretical guidance for the application of SDFs in the food industry. This paper not only provides a theoretical basis for the precise application of SDFs in functional foods but also reveals the potential mechanisms involved in regulating the structure of SDFs through processing technology to achieve nutritional intervention in metabolic diseases, which is an important guiding value for the development of food ingredients with specific health effects.

## 1. Introduction

Fruits and vegetables are rich sources of biologically active compounds beneficial to human health, including dietary fibers (DFs), vitamins, minerals, carotenoids, and polyphenols [1,2]. Current research efforts are primarily focused on developing products rich in functional compounds that impact human health, guiding consumers towards balanced dietary choices and preventing metabolic diseases like obesity, diabetes, and hypertension. Consequently, DF has garnered significant attention as the “seventh major nutrient”. Similarly to carbohydrates, DF resists digestion and absorption in the small intestine. Over the years, our understanding of DF has significantly advanced in the physiological and analytical realms. “Soluble dietary fiber (SDF), as an important component of dietary fiber, has a wide range of physiological functions, particularly demonstrating significant benefits in blood sugar regulation, gut health, and the prevention of cardiovascular diseases”. Nevertheless, the physiological effects of SDF depend largely on its source, the proportion of its components, the degree of milling of raw materials, and thermal processing. Since the concept of DF was introduced, numerous epidemiological studies have demonstrated a link between the structural and functional attributes of DF intake and the risk of certain diseases [3,4]. Recommendations emphasize a daily DF intake of 30–45 g per individual, yet current intake levels in Western countries hover around 20 g per person per day [5]. The World Health Organization recommends that adults consume at least 25 g of dietary fiber daily to promote health and prevent chronic diseases [6]. According to the Dietary Guidelines from the United States Department of Agriculture, the recommended daily intake of dietary fiber is 25 g for women and 38 g for men [7]. Canada’s dietary guidelines recommend that adults consume between 25 and 38 g of dietary fiber daily; however, the actual intake often falls short of these recommendations [8]. The content of soluble dietary fiber in dietary fibers is usually only 3% to 5% [9]. The waste of resources and the need for dietary fiber intake highlight the need for research on dietary fiber extraction. Given that the handling of food throughout processing stages may impact the final product’s SDF content and fiber types, the processing of samples plays a pivotal role [10].

Food processing transforms raw food materials into consumable products through physical or chemical means or converts one type of food into another form, crucial for ensuring or enhancing food safety and extending shelf life. Fruits and vegetables undergo various processing treatments, such as drying, heating, low-temperature treatments, fermentation, microwaving, and ultrasonication, post-harvest from the farm, where the quality of these produces is significantly impacted by the diverse processing methods employed. Understanding how food processing techniques affect the structure and activity of SDF helps in selecting appropriate technologies and conditions during food processing, thereby optimizing the utilization of food ingredients.

Currently, although research has explored the influence of different processing methods of fruits and vegetables on soluble dietary fibers, systematic studies on common and specific variations induced by different processing methods on the structural and nutritional properties of soluble dietary fiber are still lacking, limiting the rational selection of processing methods and the efficient development of SDF originating from fruits and vegetables. In view of this, this work provides a comprehensive assessment of the effects of physical processing, physical–chemical processing, and biological processing methods on the structure–nutritional features of SDF in different sources of fruits and vegetables in order to identify the commonalities and differences between processing methods in terms of improving the structural and nutritional properties of SDF. This work could guide and provide insights into the development and application of SDF in the functional food and biomedical fields.

## 2. Structural and Nutritional Properties of Soluble Dietary Fibers in Fruits and Vegetables

### 2.1. Structural Features of Soluble Dietary Fibers

**Figure 1:**
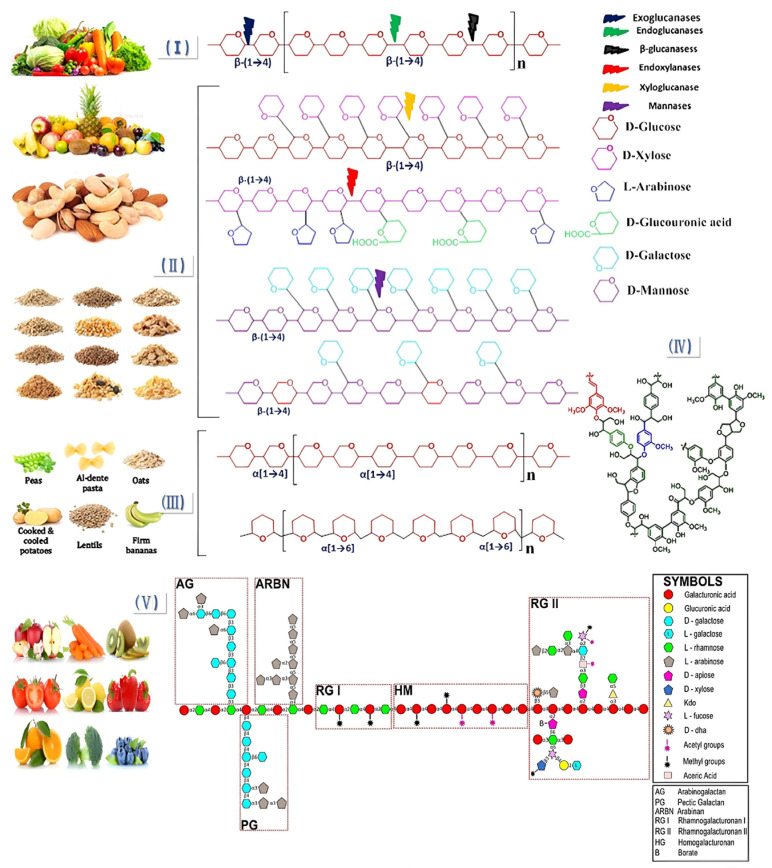
Structural diagram of DF. (I) Cellulose; (II) hemicellulose: xyloglucan, arabinoxylan glucuronate, galactomannan, and galactoglucomannan; (III) resistant starch: amylose and amylopectin; (IV) lignin; (V) pectin [22].

**Figure 2:**
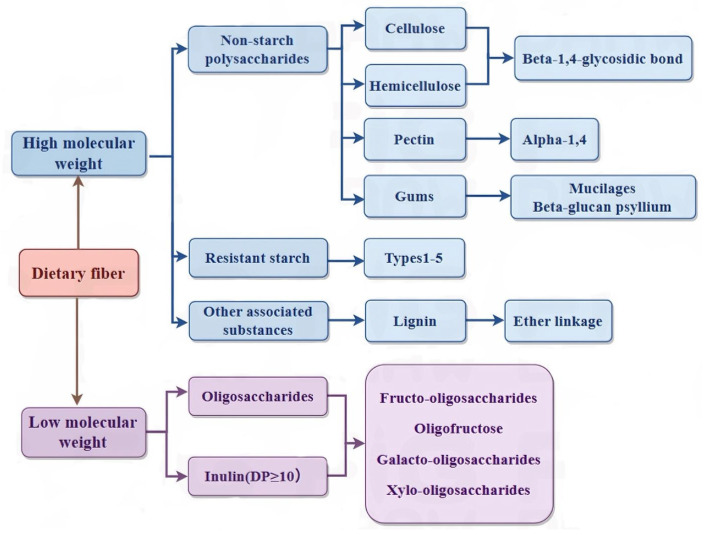
DF classification.

Based on its solubility in water, DF is classified as a soluble dietary fiber (SDF) (pectin, gum, and mucilage) or insoluble dietary fiber (IDF) (cellulose, hemicellulose, and lignin). SDF is mainly composed of pectin. Pectin, as the binding material of plant cell walls, is a complex polysaccharide (mainly composed of galacturonic acid chains), with ion groups (such as hydroxyl, methoxy, amino) as the main components. As a class of macromolecules, the structural classification of SDF follows that of proteins and nucleic acids and can be divided into primary and multilevel structures. The primary structure is complex, including the composition of sugar groups, the connection mode, the arrangement order, the length of the sugar chain, the presence or absence of branches, and the location of branches. The multilevel structure refers to the formation of regular and coarse aggregates with polysaccharides as the main chain while being combined with several functional groups such as hydroxyl, carboxyl, amino, and other functional groups [11]. SDF includes pectin, β-glucan, oligosaccharides, inulin, alginate, and fungal polysaccharides. Figure 1 shows the structure of DF. Figure 2 shows the classification of DF.

Monosaccharide composition. Monosaccharide composition analysis is an important method for studying the physicochemical properties and structure–activity relationships of polysaccharides [12]. Research has found that the main monosaccharides in the SDF of oranges are galacturonic acid and glucose, while the two monosaccharides with the highest content in grapefruits, lemons, pomelos, and citrus peels are galacturonic acid and arabinose [13]. The peel of dragon fruits is mainly composed of galacturonic acid, mannose, and xylose [14]. The main monosaccharide extracted from the by-products of sweet potatoes and purple sweet potatoes is galactose [15]. The monosaccharide composition of SDF varies due to its different sources.

Molecular weight. The solubility, viscosity, and gelation properties of SDF vary with its molecular weight. The average molecular weights of SDF in citrus, apple, and potato are 84~743, 103~485, and 2~1819 kDa, respectively [16]. It has been found that in general, the shorter the carbohydrate chain, the faster the fiber fermentation, and the fiber may have good water solubility.

Functional group structural features. SDF is rich in polar groups (such as hydroxyl and carboxyl groups), which form hydrogen bonds with water molecules and may contribute to its physicochemical and physiological properties. The FTIR spectrum reveals characteristic absorption bands of the O-H and C-H stretching vibrations at around 3440 and 2925 cm^−1^, respectively. The absorption bands at 1745 and 1630 cm^−1^ are attributed to the C=O stretching vibrations of O-acetyl groups and carboxyl ions (COO−), respectively [17]. NMR reveals that the chemical shifts in H1 in the→4)-α-Glcp-(1→main chain of α-glucan range from 5.03 to 5.33 ppm, while the chemical shifts in H1 in the →6)-α-Glcp-(1→main chain range from 4.69 to 5.08 ppm. For →3)-β-Glcp-(1→ and →3,6)-β-Glcp-(1→ residues in β-glucan, the chemical shift of H1 ranges from 4.42 ppm to 4.57 ppm, while the resonance region of C1 is 101.9–103.7 ppm [18,19]. Mannan typically consists of linear main chains of α-(1→6)-linked Man residues, with side chains of non-reducing end α-(1→2)- and α-(1→3)-linked Man residues. Functional groups are key factors determining the three-dimensional structure of SDF molecules. Here are several major functional groups and their effects on the three-dimensional structure of soluble dietary fiber: Hydroxyl groups are hydrophilic, capable of forming hydrogen bonds with water molecules, making SDF soluble in water. Hydrogen bond interactions between hydroxyl groups promote polysaccharide chains to form spiral or coiled conformations in water. Carboxyl groups are also hydrophilic, capable of forming hydrogen bonds with water molecules, and can ionize at specific pH values to generate negative charges, increasing intermolecular repulsion forces, leading to the extension of polysaccharide chains in water. The type and linkage of glycosidic bonds determine the conformation of polysaccharide chains. For example, β-1,3- and β-1,6-glycosidic bonds often lead to more linear conformations of polysaccharide chains, while α-1,4- and α-1,6-glycosidic bonds may cause chain bending. Hydrophobic groups tend to aggregate in the interior of molecules to avoid contact with water, which may result in the formation of tight folding structures in certain regions of polysaccharide chains.

Microscopic morphology. The microstructure of DF (porosity, size, shape, and particle arrangement) plays an important role in its physicochemical activity [20]. Compared to the SDF of oranges, lemons, and mandarins, the surface of grapefruit SDF is more irregular and uneven, with more pore structures [13]. The surface of SDF from potato by-products exhibited a rough, irregular, and porous morphology [15]. In addition, the porosity, size, and shape of SDF affect its ability to bind with cholesterol and bile acids.

Crystallinity analysis. The peaks of SDF were specifically analyzed by X-ray diffraction (XRD), and this study showed that edamame is treated with high hydrostatic pressure to reduce crystallinity. XRD pattern analysis revealed that broken fibers strengthened the crystal plane of fiber crystal I; a reduction in crystallinity caused the fibers to form a loose structure under thermal stability, which significantly improved water retention capacity and enhanced swelling properties [21].

### 2.2. Nutritional Characteristics of Soluble Dietary Fiber

**Figure 3:**
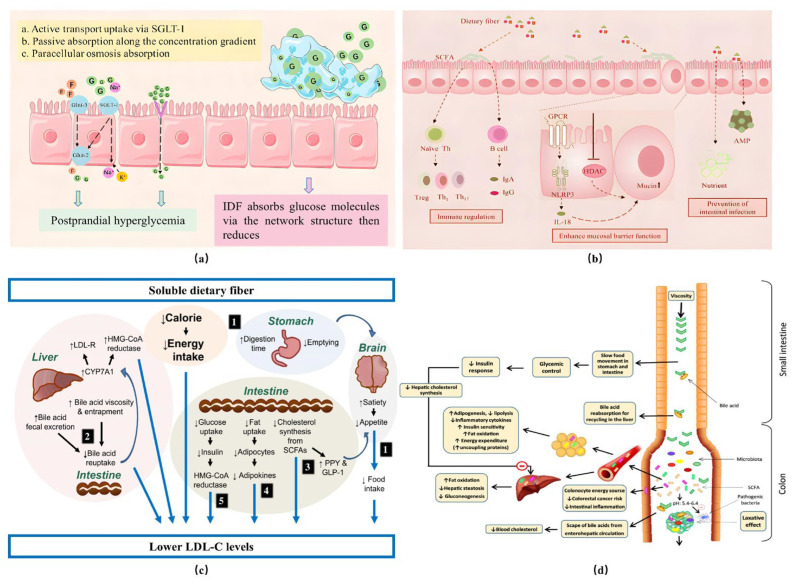
Functional properties of SDF. (**a**) Mechanism of glucose adsorption (G: glucose molecule; F: fructose; SGLT-1: sodium–glucose cotransporter; GLUT-1, GLUT-5: glucose transporter); (**b**) protective mechanism of SDF in intestinal flora. LDL-C = low-density lipoprotein cholesterol; LDL-R = low-density lipoprotein receptor; HMG-CoA = β-hydroxy-β-methylglutaryl-coenzyme A; CYP7A1 = cholesterol 7α-hydroxylase; SCFA = short-chain fatty acids; GLP-1 = glucagon-like peptide 1; (**c**) potential mechanism of lipid reduction via SDF; (**d**) mechanism of SDF in regulating appetite [25,26,27,28].

The nutritional characteristics of SDF are mainly reflected in its multiple benefits to human health, including lowering the level of blood lipids, reducing blood sugar, improving intestinal flora, and controlling weight [23], as shown in Figure 3.

Gut microbiota improvement. Fruit and vegetable SDF can change the composition of the gut microbiota and selectively stimulate the colonization and activity of bacteria in the colon, thereby affecting human health. Studies have reported that SDF can enhance the extracellular fermentation ability of the gut microbiota. SDF can be fermented and utilized by gut microbiota to reshape the gut microbiota, thereby alleviating constipation [24].

Blood glucose lowering function. Fruit and vegetable SDF can improve insulin sensitivity, increase insulin release, control postprandial blood glucose elevation, inhibit alpha-amylase activity, interfere with glucose diffusion, and delay the entry of glucose into the blood, thereby regulating blood glucose levels and improving blood glucose abnormalities.

Blood lipid lowering function. Soluble viscous fiber types affect the absorption of the small intestine by forming a gel, thereby reducing postprandial blood glucose and blood lipid levels.

Obesity prevention. SDF can also increase satiety, reduce food intake, help control weight, and prevent obesity. Current research has found that adding α-cyclodextrin to a high-fat diet can significantly reduce weight gain in rats. Increasing pectin intake can enhance satiety in rats, effectively reducing food intake, weight gain, and body fat content.

Reduction in the absorption of harmful substances. SDF can also adsorb and eliminate harmful substances in the body, such as heavy metals, cholesterol, etc., and has detoxifying and detoxifying effects.

### 2.3. Relationship Between Structural and Nutritional Properties of Soluble Dietary Fibers

**Figure 4:**
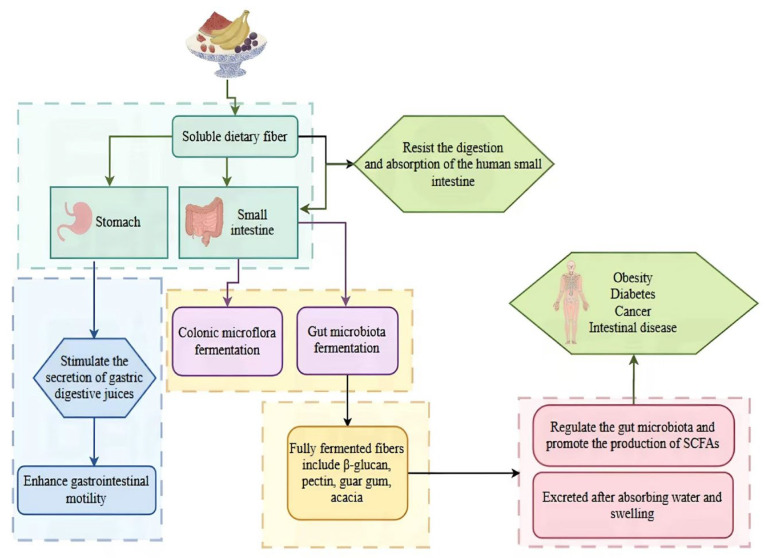
Metabolic and functional properties of SDF in vivo.

**Figure 5:**
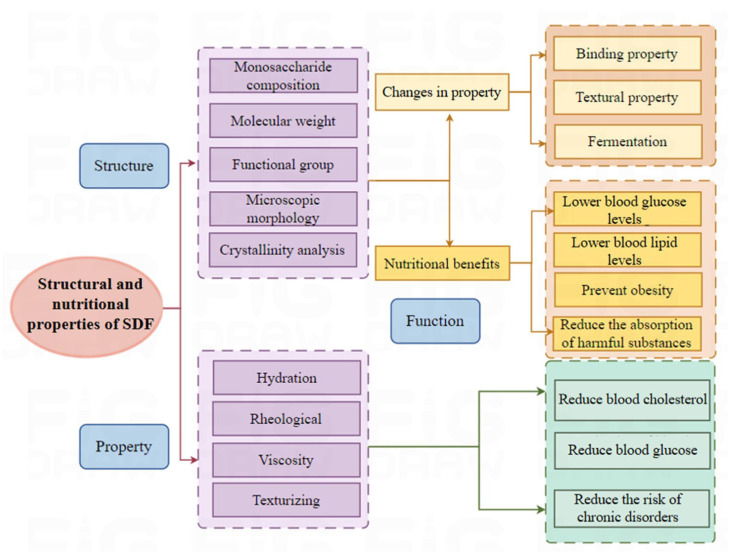
Structural, property, and functional relationships of SDF.

The structural characteristics of SDF give it unique physiological functions in the human body, playing an important role in maintaining health. Figure 4 and Figure 5, respectively, show the relationship between the metabolic and functional properties of SDF in vivo and its structure, properties, and functions.

(1) The chemical composition of SDF affects the prevention of obesity. The main soluble dietary fibers in fruits and vegetables are pectin and inulin, which are highly viscous and strongly absorbent and can increase satiety and reduce energy intake. In addition, there are findings that indicate that soluble dietary fiber improves energy homeostasis and prevents obesity by increasing the diversity of the gut microbiota and the colonization of beneficial bacteria [29].
(2) The molecular weight of SDF affects the absorption of harmful substances. SDFs with different molecular weights exhibit different functions. Studies have found that plantago polysaccharides with medium molecular weight show the strongest immune regulatory activity. In type 2 diabetic rats, the effects of medium-molecular-weight (252~757 kDa) konjac glucomannan on reducing fasting blood glucose, total cholesterol, and low-density lipoprotein cholesterol levels are superior to those of higher- or lower-molecular-weight konjac glucomannan. In addition, dietary polysaccharides with relatively low molecular weight often have good hydration properties, which allow SDF to form a gel-like substance in the intestine, helping to increase fecal volume and soften stool.
(3) The monosaccharide composition of SDF affects the gut microbiota. Common monosaccharides in SDF include Glc, Xyl, Ara, Rha, Gal, and Man, which are mainly linked by β-1,4-glycosidic bonds and α-1,4-glycosidic bonds [30]. Studies have found that different monosaccharide compositions affect the fermentability of fiber. Some dietary fibers containing fructan structures (e.g., fructo-oligosaccharides and inulin) are highly susceptible to fermentation via beneficial gut microbiota. Additionally, the composition and ratio of different monosaccharides can affect the species of microbes in the gut; for example, galactose and lactose may be more favorable for the growth of specific beneficial bacteria. The growth of these probiotic bacteria can improve the balance of gut microbiota, enhance gut barrier function, and reduce the risk of intestinal diseases.
(4) The surface properties of SDF affect blood sugar and blood lipid levels. The surface properties of SDF (such as hydrophilicity and hydrophobicity) affect its interactions with water and other nutrients. Research has found that hydrophilic fibers can better absorb water, increasing the volume of intestinal contents. In addition, the particle size, surface area, and average particle size of SDF particles all have a certain impact on the water retention capacity (WRC), water holding capacity (WHC), oil holding capacity (OHC), and swelling capacity (SC) of SDF. However, excessively small particle sizes are detrimental to improving the performance of WHC and OHC. A smaller average particle size of DF leads to higher viscosity values. Smaller fiber particle sizes provide a larger specific surface area and packing density, imparting the fluid structure and a larger amount of fiber with flow resistance. Compared to non-viscous fiber, high-viscosity fiber can increase intestinal viscosity, delay gastric emptying, and reduce the rate of glucose absorption, thereby weakening postprandial glucose and insulin responses [31].

## 3. Effects of Drying Processing Methods on Soluble Dietary Fiber in Fruits and Vegetables

**Figure 6:**
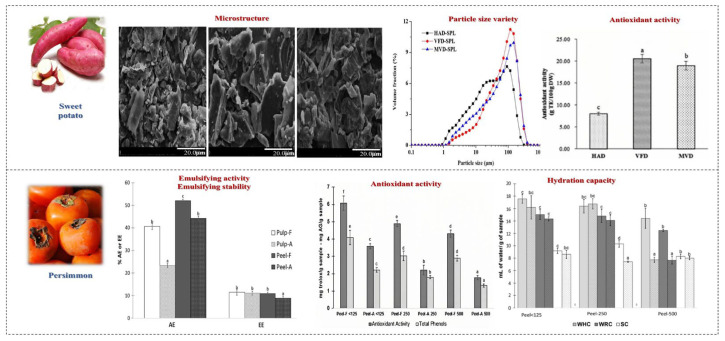
The effects of drying processing methods on SDF in fruits and vegetables. Different letters mean significant difference (*p* < 0.05) [38,39].

Fruits and vegetables serve as the primary sources of SDF in consumer foods and also act as raw materials for producing fiber-rich products. In the processing of plant-based foods, various unit operations involving product sensory quality, nutritional quality, safety, and stability can significantly influence the structure, physicochemical properties, and nutritional value of SDF [32]. The physiological functions of SDF are attributed to its physicochemical properties, such as water solubility, water retention, swelling, and rheological behavior. Fresh food raw materials or their semi-finished products typically have a high water content. Removing this water can reduce packaging, storage, and transportation costs, making it a traditional method of food preservation. In the processing of fruits and vegetables, drying methods are commonly used to prepare dried products such as fruit and vegetable chips, which primarily include hot air drying, vacuum freeze drying, natural drying, and microwave drying. Food dehydration involves two processes, concentration and drying, and different drying conditions affect the physicochemical and functional properties of SDF in different ways, so choosing the appropriate drying method is important to optimize fiber quality. Table 1 and Figure 6, respectively, show the effects of different drying methods on the properties of SDF in fruits and vegetables.

**Table 1:** Effects of different drying methods on properties of SDF in fruits and vegetables.

Ingredients	Drying Condition	Effect on the Properties of SDF	References
Orange peel	Hot air drying, microwave drying	Water retention capacity and color were not affected, the rehydration performance of the fiber was changed, and the swelling capacity was increased.	[33]
Peach	Microwave drying	A lower drying temperature led to higher hydraulic capacity, oil capacity, expansion force, and specific volume.	[34]
Orange peel	Freeze drying, hot air drying	Freeze drying increased the viscosity of SDF and led to a higher glucose adsorption capacity and glucose dialysis block index. Hot air drying reduced the molecular weight of SDF.	[35]
Carrot	Hot air drying	Water retention, total phenolic content, and antioxidant activity markedly decreased.	[36]
Cabbage	Vacuum drying	Vacuum drying temperature had no effect on the total phenolic content or antioxidant capacity of SDF.	[37]

### 3.1. Hot Air Drying

Hot air drying is one of the mainstream technologies used for fruit and vegetable processing, and processed fruit and vegetable crisps and other products are favored by consumers. The hot air drying technique is based on the principles of mass and heat transfer, using a heat source to provide heat. By using a fan, hot air is blown into an oven or drying chamber, transferring the heat from the drying medium to the material, causing the surface moisture of the product to be heated and vaporized into water vapor, which then diffuses into the surrounding air.

Hot air drying can improve the structure and nutritional properties of DF by altering the ratio of soluble and insoluble fibers in fruits and vegetables. High temperatures can change the structure of DF in fruits and vegetables, facilitating the conversion of IDF to SDF, making it more crisp and easier to dissolve, thereby affecting the taste and texture of fruit and vegetable crisps. Liu et al. investigated the effects of freeze drying, hot air drying, and vacuum drying on the physicochemical properties of soluble and insoluble dietary fiber from orange peels, showing that hot air drying led to a decrease in the molecular weight of soluble dietary fiber compared with that obtained when freeze drying [35]. According to Svanberg et al., the effects of different processing methods on the DF content of fruits and vegetables present complex variation characteristics. For example, the degree of depolymerization and the intermolecular association of water-soluble polysaccharides in carrots after heat treatment depend on the degree of heat treatment.

The physical and chemical properties of SDF are influenced by factors such as drying temperature and drying time. For physicochemical properties, high-temperature drying treatment leads to the depolymerization of DF into soluble fractions and alters its hydration properties and fat adsorption capacity. Studies have shown that orange peel samples have a higher swelling value of SDF at a drying temperature around 50 °C, but as the drying temperature increases, the water retention capacity and solubility of SDF decrease significantly. Talens et al. used the by-products of orange processing as raw materials, and a new fiber raw material was obtained by the method of hot air and microwave coupled drying. Compared with those obtained when using hot air drying, processing time (92%) and energy consumption (77%) were significantly reduced. Drying treatment does not affect the chemical composition, water holding capacity, or color of orange fibers compared with hot air drying. The total dietary fiber is close to 0.6 kg/kg, with SDF: IDF at a 1:1 ratio. The shrinkage–expansion phenomenon occurring during drying affects the chemical potential gradient of water, altering the fiber’s rehydration properties. An increase in particle size enhances the swelling capacity of fiber during hydration [33]. Garau found that different drying temperatures led to a reduction in water retention capacity, swelling capacity, and fat absorption ability, while higher drying temperatures also diminished the antioxidant capacity of DF from carrot skin [36].

The quality and antioxidant capacity of SDF are affected by drying temperature and time. When the drying time is prolonged or a higher drying temperature is used, the components and antioxidant activity of SDF are degraded. Overall, hot air drying can lead to the losses of certain substances or active ingredients in fruits and vegetables. In addition, the high temperature used during hot air drying can lead to the Maillard reaction, resulting in browning and the deepening of the color of SDF, thus limiting its application. Therefore, it is essential to consider the processing history of SDF concentrates and their associated bioactive compounds to facilitate a more effective evaluation before their incorporation into various foods (functional foods) or as dietary supplements.

### 3.2. Freeze Drying

Freeze drying is a process that sublimates the moisture from frozen samples using vacuum and rapid freezing technology. This method can reduce the residual moisture content of a material to below 1%. The working pressure used during the initial drying phase is usually between 0.5 hPa and 10^−2^ hPa, and it can reach up to 10^−3^ hPa during the secondary drying phase or when adjusting the chamber. The primary purpose of freeze drying is to lower the moisture content of fruit powder, extend its shelf life, reduce product size, decrease transportation costs, and enhance the nutritional value and taste of food. Okra, blueberries, peaches, apple slices, mushrooms, bananas, red dates, durian, carrots, etc., obtained through vacuum freeze drying technology have been proven to have good color, form a loose porous structure after drying, and maintain the original physical characteristics of the material well. The retention rate of nutrients such as polyphenols, flavonoids, and vitamin C in the materials reaches over 90% [40,41,42].

Freeze drying can better preserve the content of DF compared to other drying methods, significantly reducing the bulk density of SDF and IDF. The solids obtained by freeze drying retain the sensory characteristics of fresh fruits and do not exhibit any “off-flavors”; therefore, their taste and aroma activity values are the highest. Ara et al. found that freeze-dried guava had higher IDF and TDF content but lower SDF content [43]. The pectin content in passion fruit peel after freeze drying was higher than the pectin content in fresh residues, indicating that the release of this type of fiber was promoted during the drying and dehydration process, and long-term freeze drying can promote the preservation of this fiber [44]. Liu reported that the DF particles obtained from freeze-dried orange peels had a more irregular and uneven surface, a relatively loose structure, and a higher number of cracks and voids; however, the matrix structure of DF hardly shrank during the freeze drying process [35]. The results showed that the antioxidant activity of persimmon peel fibers was greater than that of lemon, orange, and peach fibers [39]. In summary, it can be speculated that freeze drying preserves the quality of SDF more effectively than other drying methods. Based on these findings, it is recommended that freeze drying be utilized in the processing of DF to produce high-quality products for functional foods.

### 3.3. Microwave Drying

Microwave drying is mainly used for making dried fruits and vegetables, resulting in the better preservation of their color, flavor, and nutritional content. The principle of this method involves applying microwave radiation to the material to be dried, and under the high-speed rotation of the microwave, polar molecules such as water in the material rotate rapidly, generating heat through friction and causing the temperature to rise. As a result, a significant number of water molecules evaporate from the material, leading to a reduction in its moisture content and achieving the desired drying effect [45].

Microwave treatment influences the composition of DF, increasing the content of SDF while simultaneously reducing the levels of fat, protein, and other impurities [46]. Furthermore, microwave drying affects the functional properties of SDF, enhancing its water retention capacity, oil retention capacity, and fermentation rate [47]. The impact of microwave treatment on the hydration ability of SDF mainly comes from three aspects: microwave drying can increase thermal conductivity, producing an expansion effect and making the internal structure of fruits and vegetables SDF more loose, thereby increasing water holding capacity and expansion ability; microwave drying induces moderate structural segregation that promotes pectin solubilization, but once it exceeds a certain limit, it leads to pectin degradation; and an uneven surface of fruits and vegetables causes localized high temperatures during drying, resulting in partial charring, leading to a decrease in the hydration ability of SDF. The combined effect of these three factors results in the relatively low hydration ability of fruits and vegetables SDF after microwave drying, which in turn has a significant impact on the microstructure of SDF at high temperatures. Thus, the influence of microwave treatment on fruit and vegetable SDF is relatively small. Although microwave drying demonstrates moderate performance in preserving nutritional components and functional properties, the high physicochemical properties and drying rate and low cost indicate that microwave drying has greater potential as a new drying method.

Overall, hot air drying, freeze drying, and microwave drying are three common techniques for processing fruit and vegetable dietary fiber, each of which has different effects on the structure and nutritional composition of dietary fiber. Hot air drying may lead to a decrease in the total content of dietary fiber and affect its hydration characteristics and expansibility by changing the structure and proportion of dietary fiber, especially impacting soluble and insoluble dietary fiber. High-temperature drying may also destroy heat-sensitive components, reducing the antioxidant capacity of soluble dietary fiber. However, by optimizing temperature and time, the effects of hot air drying can be improved, especially when combined with other drying methods such as microwave drying, to enhance the functionality of dietary fiber. In contrast, freeze-drying removes moisture through sublimation under low-temperature and vacuum conditions,, better preserving the structure and functionality of dietary fiber, especially in terms of hydration, expansibility, and emulsification performance. After freeze drying, dietary fiber presents a loose and porous structure, which not only increases its specific surface area and molecular weight but also allows it to maintain a lower bulk density, significantly improving its water absorption and oil adsorption capacity. Additionally, freeze drying effectively protects specific dietary fiber components in fruit peel, such as pectin, enhancing their functionality. Overall, freeze drying has significant advantages in preserving dietary fiber and its nutritional components, especially suitable for temperature-sensitive nutrients. On the other hand, hot air drying is more suitable for large-scale production but requires the optimization of drying conditions based on specific products. Microwave drying, as an efficient and fast drying method, can effectively enhance the functional characteristics of dietary fiber, especially in improving fermentability. The combination of different drying methods can maximize the retention of the nutritional and functional properties of dietary fiber while improving drying efficiency.

## 4. Effects of Heating Processing Methods on Soluble Dietary Fiber in Fruits and Vegetables

Heat treatment significantly influences the structural and nutritional properties of soluble dietary fiber during fruit and vegetable processing. Thermal processing, including blanching, sterilization, and extrusion, induces modifications in SDF’s molecular structure, such as depolymerization, changes in glycosidic linkages, and the exposure of functional groups—which subsequently alter its hydration capacity, viscosity, and fermentability. While excessive heating may degrade SDF and disrupt its physiological benefits, controlled thermal treatment can enhance its bioactivity, including antioxidant capacity and prebiotic potential. The extent of these changes depends on processing parameters, highlighting the need for optimized thermal protocols to maximize SDF’s nutritional and functional value.

### 4.1. Blanching Treatment

Blanching primarily aimed at inactivating endogenous enzymes to prevent enzymatic browning and nutrient degradation. This process is primarily employed to preserve the original quality of food and to prevent or reduce the degradation of its color, aroma, flavor, and nutritional value. Blanching is mainly applied to vegetables and certain fruits, typically as a pretreatment prior to freezing, drying, or preserving. Recent studies indicate that high temperatures can break covalent bonds and disrupt the physical structure of macromolecules, leading to alterations in their functions. Current research on the effects of heat treatment on fruits and vegetables mainly focuses on changes in proteins and starch, with little attention paid to changes in the SDF of fruits and vegetables [48,49,50].

Bleaching treatment significantly changes the composition and nutritional properties of dietary fibers in fruits and vegetables through short-term thermal action in enzymatic sterilization and selectively affects heat-sensitive nutrients. It has been found that using bleaching to treat sweet corn resulted in a slight increase in SDF content, did not significantly change the concentration of total monosaccharides, and effectively retained ketoses [51]. Namiko Nishidad et al. used low-temperature blanching (LTB) followed by high-temperature blanching (HTB) pretreatment to prevent the excessive softening of frozen Japanese radish. The results suggest that LTB treatment promoted the demethylation of pectin, leading to strong cell wall adhesion, and prevented the depolymerization of pectin by the subsequent HTB treatment [52]. Blanching treatment enhanced the calorie, carbohydrate, and vitamin A content of mangifera odorata L. fruit peel. However, it reduced the content of vitamin C and dietary fiber in the pericarp [53]. Bleaching treatment can optimize the ratio of dietary fiber but inevitably leads to heat-sensitive vitamin loss, highlighting the necessity of precise process control to achieve an optimal balance between nutrient retention and functional improvement

### 4.2. Sterilization Treatment

Fruits and vegetables are mostly consumed as primary agricultural products, but some are processed into value-added products like juice, puree, jam, jelly, etc. Nevertheless, fruit and vegetable juices have abundant nutritional constituents and water content, making them prone to spoilage due to microbial contamination and enzyme-catalyzed reactions, which become limiting factors in processing and preservation. Fruit and vegetable products are prone to spoilage; therefore, using an efficient sterilization method is particularly important. There are many methods of sterilizing fruit and vegetable juices, and this section mainly introduces the concept of heat sterilization.

Pasteurization is a process that involves applying heat for a specific duration to eliminate harmful organisms and deactivate relevant enzymes, thereby extending the shelf life of fruit and vegetable juices. While the nutritional composition of fruits and vegetables is altered during high-temperature pasteurization, the active ingredients remain largely unaffected. However, the structural and nutritional properties of proteins, sugars, and SDF undergo significant changes [54]. The high-temperature pasteurization process can cause changes in the microstructure of DFs in fruits and vegetables, leading to varying degrees of changes in SDF and IDF. Different research results have been reported for fruit and vegetable products processed by high-temperature pasteurization. The TDF content in mango puree remained unchanged after high-temperature pasteurization treatment [55]. On the other hand, the proportion of SDF in persimmon puree exhibited significant changes after high-temperature pasteurization treatment. With increasing processing time and pressure levels, the IDF content increased to 13%, and the SDF content of persimmons decreased by 27% under pressure conditions of 400 MPa/min at ambient temperature [56]. In addition, pasteurization can lead to cellulose loss and affect the physicochemical properties of DF and changes in pectin. Studies have found that sterilization reduces IDF content in onions while increasing SDF content, thereby improving the ratio of the two; the swelling capacity of DFs decreases, while the water holding capacity shows no significant change [57].

### 4.3. Expansion Processing Treatment

#### 4.3.1. Extrusion Puffing

Extrusion puffing is an efficient and fast food processing technology based on mixing. In food processing, screw extruders are an example of commonly used extrusion puffing equipment. Currently, various types of extrusion puffing machines exist, which can be categorized into single-screw, twin-screw, and multi-screw extrusion puffing presses based on the number of screws utilized. Through the synergistic effects of high shear force and high temperature and pressure, extrusion puffing technology can effectively break the glycosidic and hydrogen bonds of the insoluble dietary fiber (IDF) in the dietary fiber (DF) of fruits and vegetables, thereby converting them into easily digestible, water-soluble, low-molecular-weight polysaccharides. Furthermore, extrusion puffing can cause the rapid evaporation of moisture from DF at the extruder outlet, altering the spatial structures both between and within molecules, thereby modifying the chemical properties of DF.

Extrusion puffing can promote the mutual transformation of two types of DFs, with some IDF in fruits and vegetables transforming into lower-molecular-weight SDF, reducing the molecular weight of pectin and hemicellulose, which can improve the functionality and texture of food. The reason for this is that under high-temperature, -pressure, and -shear conditions, fibers will break and degrade, resulting in a change in the polarity of molecules. In addition, conducting extrusion operations under low moisture content significantly increases the SDF content [58]. At the same time, different process parameters of extrusion puffing equipment have an impact on the content of DFs. Guo et al. found that the content of SDF in garlic skin increased from 5.31 ± 0.58% to 15.87 ± 0.88% at 170 °C [59]. Observations made by Guo et al. using scanning electron microscopy (SEM) revealed that the surface of untreated SDF was smoother with a larger pore structure, while the extruded SDF appeared denser with larger pores [59].

Extrusion can modify various physicochemical properties and physiological activities, such as water retention capacity, oil retention capacity, expansion power, and cholesterol adsorption capacity. The reason for this lies in the fact that cellulose and lignin undergo molecular bond breakages under high temperature, high pressure, and high shear force, leading to changes in molecular polarity and chemical and biochemical properties, when converted to soluble dietary fiber. In their study, Xue et al. found that optimizing extrusion conditions significantly increased the water holding capacity, oil holding capacity, swelling power, glucose absorption coefficient, and bile acid absorption coefficient of mushroom residue DF [60]. Redgwell et al. reported that all extruded fibers exhibited a reduced water retention capacity compared to citrus-based fibers, a phenomenon attributed to the destruction of the intact cell wall structure during the extrusion process [61]. Huang et al. conducted several in vitro experiments to determine the indicators predicting the hypoglycemic, hypocholesterolemic, and fermentative abilities of squeezed orange peels. The results showed that compared with orange peel, squeezed orange peel effectively delayed glucose diffusion and inhibited α-amylase activity. Moreover, squeezed orange peel demonstrated the ability to bind with cholesterol micelles and bile acids. During the fermentation process of squeezed orange peel, high levels of short-chain fatty acids were produced [62].

Although extrusion puffing processing technology is efficient and has a high retention rate of heat-sensitive nutrients, there are still problems with this processing technology, such as the quality of SDF being greatly affected by process parameters, the unclear mechanisms of SDF in materials, high equipment costs, and easy loss during use. Therefore, future development efforts should focus on improving extrusion puffing technology and also explore the best production conditions based on different sources of SDF.

#### 4.3.2. Steam Explosion

**Figure 7:**
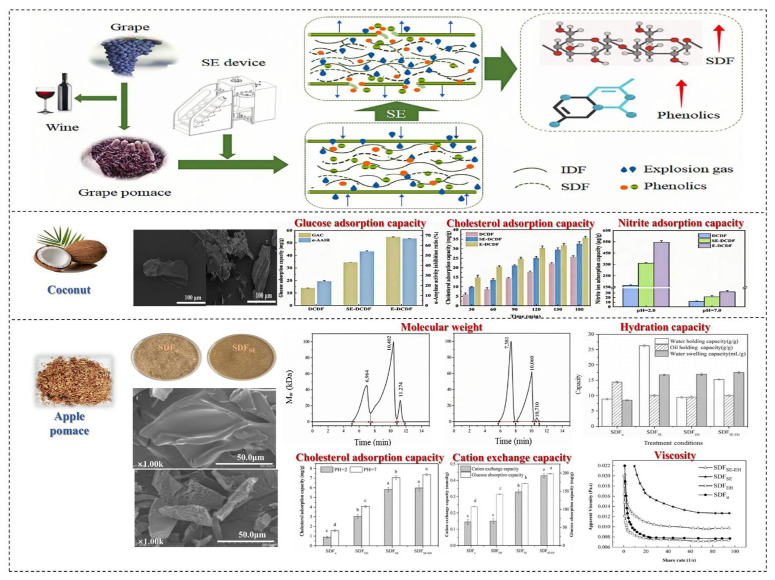
The effects of drying processing methods on SDF in fruits and vegetables. Different letters mean significant difference (*p* < 0.05) [65,66,67].

Wet extrusion puffing technology mainly involves the introduction of steam explosion (SE) technology. The principle of SE technology involves introducing high-temperature and high-pressure steam into raw materials for cooking, allowing the steam to fully penetrate the organization and cells of the material and then release high pressure instantly in milliseconds. During this process, the moisture within the sample quickly expands and escapes, generating significant shear force, which disrupts the fiber structure and chemical composition of the raw materials, thereby enhancing the properties of the fibers [63]. SE technology significantly affects the structure of DFs. During high-temperature and high-pressure cooking, the acid hydrolysis of water under these conditions promotes the hydrolysis of acetyl groups in hemicellulose. During pressure release, mechanical forces cause fiber breakage, leading to the degradation of cellulose and hemicellulose [64]. Figure 7 shows the effects of drying processing methods on SDF in fruits and vegetables.

The use of SE technology leads to the degradation of IDF and its transformation into SDF through explosive shearing and steam hydrolysis, and with a further increase in SE pressure and holding time, the content of DF first increases and then decreases [68]. Cui et al. reported that the extraction rate of SDF in grape pomace increased with the concentration of SE, ranging from 7.25 ± 0.61 g/100 g to 14.76 ± 1.37 g/100 g. Under a pressure of 1.2 MPa, the extraction yield of SDF was the highest, demonstrating a 2.04 times increase compared to the untreated control group... In addition, SE significantly reduced the yield of IDF, which decreased from 71.83 ± 2.98 g/100 g to 56.19 ± 1.24 g/100 g. IDF in grape pomace was converted to SDF, which enhanced the oil holding capacity and sodium nitrite binding capacity of SDF [65]. SE technology disrupts the compact structure of DF, increasing its specific surface area and stability. The surface of DF without SE treatment is smooth and compact, but after SE treatment, a lot of cavities and pores appear on the surface of DF, suggesting that SE treatment damages the cell wall of the raw materials, leaches out small molecular substances inside the raw materials, and reduces the degree of fiber polymerization, but it does not affect the crystal structure of DF. For example, the infrared spectrum of sweet potato after SE is roughly similar, but some large molecular cellulose is transformed into small molecular sugars. Wang et al. investigated silver ear modification treatment that was conducted using steam explosion. Their research found that the extraction rate of SDF increased by 1.42 times (from 23.33 ± 0.42% to 33.21 ± 0.28%) [66]. Steam explosion (SE) disrupted the dense structure of SDF, increasing its specific surface area and thermal stability. Additionally, the structural changes induced by SE led to improved functional properties, with SDF exhibiting better hydration properties (water holding capacity, oil holding capacity, and swelling capacity increased by 1.23, 1.59, and 1.24 times, respectively) and blood glucose lowering performance (glucose adsorption capacity increased by 1.84 times under 100 mmol/L glucose) [66].

The physicochemical properties of SDF, including water holding capacity, oil holding capacity, and expansion ability, directly affect its ability to combine and interact with other components during food processing, such as extrusion, mixing, and homogenization, thereby influencing the texture and quality of food [69]. SE increases the water holding capacity, oil holding capacity, and swelling capacity of SDF by 1.23, 1.59, and 1.24 times, respectively. As water holding capacity and swelling capacity can alter the viscosity of food, they affect sensory properties. The oil holding capacity of DF is closely related to its surface structure; theoretically, the larger the specific surface area of particles, the stronger their ability to bind oil molecules. It was found that after microwave explosion treatment, the oil holding capacity of IDF from prickly pear pomace increased from 7.29 ± 0.68 g/g to 9.62 ± 0.66 g/g, an increase of 0.3 times; the oil holding capacity of SDF increased from 1.53 ± 0.51 g/g to 5.96 ± 0.49 g/g, an increase of 2.9 times. Moreover, SDF exhibits various biological activities, including hypoglycemic, cholesterol-lowering, and prebiotic effects. In another study, Liu et al. observed that SE pretreatment can enhance the content of PC and slightly alter the physical and chemical properties of AGP [70]. Additionally, SE pretreated polysaccharides demonstrated significantly higher α-glucosidase inhibitory activity compared to those without SE pretreatment, indicating their potential as effective α-glucosidase inhibitors. Furthermore, SE treatment facilitated the extraction of fiber components with potential prebiotic and high antioxidant activities from asparagus by-products [71].

#### 4.3.3. Pressure Difference Expansion

Pressure differential flash drying, also known as pressure differential expansion, is a drying method that involves applying high temperature and pressure to the surface of food in an instant, causing water to rapidly evaporate from food. It is used in the production of fruit and vegetable crisps. In recent years, domestic and foreign scholars have mainly focused on researching pressure differential flash drying technology based on different agricultural products, using this technology for texture modification, sterilization, the disinfection of materials, the drying of fruit trees, and the modification of macromolecules. Currently, most scholars focus on texture modification and the drying of fruits and vegetables, studying the effects of drying conditions on the quality of fruits and vegetables. The most commonly studied puffed fruit and vegetable materials include apples, sweet potatoes, carrots, peaches, and dragon fruit.

The high-temperature, short-duration conditions of pressure differential flash drying induce complex modifications in SDF through two primary ways: the thermal degradation of heat-labile polysaccharides and the physical loss of low-molecular-weight fractions via steam entrainment. While these processes may reduce total SDF yield, the remaining SDF undergoes significant structural reorganization that enhances its nutritional properties. The rapid thermo-mechanical action disrupts cell wall matrices, increasing solubility by exposing hydrophilic groups and generating smaller, more bioavailable fragments. This structural modification leads to improved hydration capacity, colloidal stability, and fermentability. Overall, extrusion puffing, steam explosion, and pressure difference expansion are all processing technologies for various dietary fibers that can significantly affect the structure and functionality of dietary fibers. By using different processing conditions, such as high temperature, high pressure, and high shear force, the molecular structure of dietary fibers can be changed, enhancing their water solubility, water holding capacity, oil holding capacity, and expansibility, thereby improving the physiological functions of dietary fibers. For example, extrusion puffing breaks down the high-molecular-weight IDF found in plant cell walls, converting them into low-molecular-weight SDF, enhancing their digestibility and water solubility while improving taste and bioavailability. Steam explosion technology can change the structure of fibers to enhance their water absorption, oil absorption, and antioxidant capacity, showing great potential in health functions, especially in blood sugar reduction and cholesterol reduction. During the pressure differential flash steaming drying process, the structure of soluble dietary fibers is damaged, leading to a decrease in their total content, but this treatment also enhances the solubility and hydration capacity of soluble dietary fibers, thereby improving their physiological activity. Therefore, despite the potential for partial degradation of SDF under certain processing technologies, optimized processing parameters can synergistically enhance its nutritional functionality,, providing broad prospects for the development of healthy foods.

## 5. Effects of Powder Processing Methods on Soluble Dietary Fiber in Fruits and Vegetables

**Figure 8:**
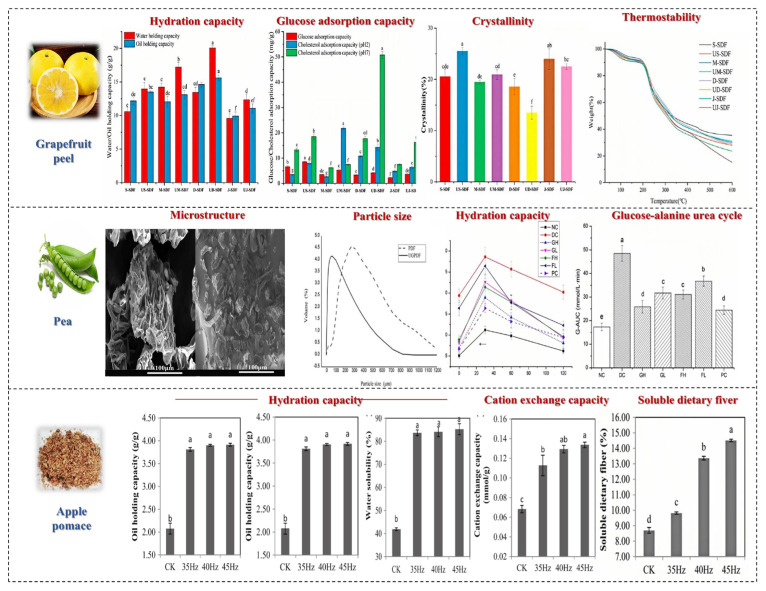
The effects of powder processing methods on SDF in fruits and vegetables. Different letters mean significant difference (*p* < 0.05) [72,73,74].

The main processing technologies used for powder raw materials include ultrafine grinding, fine classification, and surface modification, among which the most common is ultrafine grinding technology. This technology is used to crush fruit and vegetable powders into small particles of 10~25 um through mechanical or fluid dynamic forces. During the crushing process, the mechanical forces change the structure and physicochemical properties of the SDF in fruits and vegetables. Ultrafine grinding increases the specific surface area of cellulose, thereby increasing the porosity and capillary attraction of cellulose to some extent, enhancing its physicochemical properties. Conversely, as the grinding time is extended, the material experiences intense mechanical shear forces, leading to decreased water and oil retention capacities due to the damage to the fiber structure. Figure 8 shows the effects of powder processing methods on SDF in fruits and vegetables.

In the ultrafine grinding process, the main cause of an increase or decrease in molecular weight is the fracture of chemical bonds, molecular fracture, and aggregation. Ultrafine grinding technology can increase the SDF content in fruits and vegetables. Yan et al. reported that ultrafine grinding can enhance the SDF content in pear residue [75]. Qin et al. processed grapefruit peel and compared the structure and absorption properties of SDF. They found that the ultrafine grinding process disrupted the chemical bond between molecules, thereby reducing the crystallinity of SDF.

Additionally, ultrafine grinding can reduce the coarse texture of fruit and vegetable powders, thereby improving the palatability of DF. Chau reported that ultrafine grinding (including ball milling, jet milling, and high-pressure crushing) could enhance the oil retention, water retention, and swelling capacities of carrot DF [76]. Conversely, Zhu reported that the destruction of the DF matrix structure led to decreased water retention and swelling capacities [77].

Currently, research on the ultrafine grinding of fruits and vegetables mainly focuses on physicochemical properties and antioxidant aspects, with little attention paid to its functional properties, such as the impact of ultrafine grinding on blood sugar reduction. It has been demonstrated that the superfine grinding of ginger can improve the fluidity, water retention, and water solubility of its protein, thus enhancing the bioavailability of nutrients and enabling the development of functional ginger products [78]. In addition, ultrafine grinding also has a certain impact on the physiological activity of SDF. Yu et al. found that ultrafine grinding significantly increased the total antioxidant activity of SDF, the ability to scavenge DPPH free radicals, and the antioxidant capacity in linoleic acid systems. Zhang et al.’s study showed that mushroom powder prepared via mechanical and jet milling exhibited higher values in terms of SDF content, surface area, bulk density, and the water solubility index, making it suitable for instant food production [79]. Zhang et al. also demonstrated that preparing ultrafine papaya powder using a planetary ball mill could improve the processing characteristics of the powder [80]. Zhu et al. discovered that the ultrafine grinding of bitter melon powder led to higher anti-diabetic activity, reducing fasting blood sugar levels, and this powder could be used as a functional food to alleviate diabetes symptoms [81]. Research has found that compared to other processing methods, fermentation processing has a more environmentally friendly impact on the soluble dietary fiber of fruits and vegetables. The physicochemical properties of the dietary fiber obtained, such as water holding capacity, oil holding capacity, expansion, antioxidant properties, etc., are superior to those obtained using other processing methods. The SDF obtained through fermentation processing can be used as a dietary supplement added to various foods, giving the food better sensory quality and nutritional value.

Overall, ultrafine grinding can reduce the particle size of and increase the specific surface area, porosity, and capillary attraction of dietary fiber, significantly improving its water solubility, dispersibility, and adsorption, thereby enhancing its potential application in healthy foods. Ultrafine grinding may also lead to the destruction of the molecular structure of dietary fiber, affecting its water holding and oil holding capacities and other physicochemical properties. In comparison, fermentation processing increases the content of soluble dietary fiber in dietary fiber through the fermentation process, improving its adsorption, hydration ability, and ion exchange properties, thereby enhancing its functions in diabetes, cardiovascular disease prevention, and intestinal health regulation. The combination of these two processing methods can further optimize the physiological activities and health effects of fruit and vegetable dietary fiber, opening up broad prospects for its application in the food, nutrition, and health fields.

## 6. Effects of Fermenting Processing Methods on Soluble Dietary Fiber in Fruits and Vegetables

**Figure 9:**
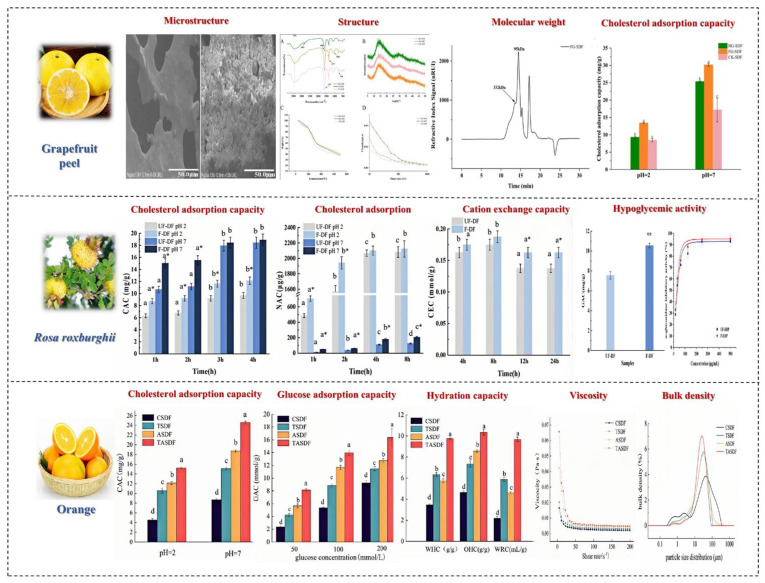
The effects of fermentation processing methods on SDF in fruits and vegetables. Different letters mean significant difference (*p* < 0.05); * Superscript asterisk on the bar mean significant difference at the same treatment time (*p* < 0.05) [82,83,84].

Fermented fruit and vegetable juice refers to a type of beverage made from one or more fresh fruits, vegetables, mushrooms, and Chinese herbs, fermented by various or single lactic acid bacteria. Microorganism fermentation is used to extract the essence of natural fruits and vegetables, concentrating organic acids, functional oligosaccharides, peptides, and other functional components produced by microbial metabolism. Studies have found that fermented fruit and vegetable juice has health benefits such as regulating intestinal flora, enhancing immunity, and eliminating free radicals. DF is a substance that is not easily absorbed by human digestive enzymes. It can be decomposed by intestinal microorganisms to produce acetic acid, propionic acid, butyric acid, and other beneficial fatty acids, lowering the pH in the colon, promoting the growth of beneficial bacteria, inhibiting the reproduction of harmful bacteria, preventing intestinal mucosal damage, and maintaining the balance of the intestinal microecosystem. Figure 9 shows the effects of fermentation methods on SDF in fruits and vegetables.

Fermented fruit and vegetable DF can increase the content of SDF and reduce the content of IDF. There are many ways in which different fermentation strains can affect fiber content, including fiber degradation, metabolic by-products, bacterial growth, and synergistic effects. By selecting appropriate strains and optimizing fermentation conditions, an optimal increase in fruit and vegetable SDF content can be achieved.

Currently, research on the physiological functions of fermented SDF mainly focuses on the following aspects. The preventive ability of fermented SDF on diabetes is significantly enhanced, as the large molecular structure is broken down by enzymes and acids produced by microorganisms, leading to increased surface area, more pores, and the formation of more SDF. This exposes more hydrogen bonds and dipoles, increasing the contact area with water, glucose, and other substances. Cui et al. dministered fermentation-modified apricot pulp SDF to rats via gavag. After 28 days of administration, the blood glucose levels in rats were significantly lower than those in the blank control group [85]. The extensively networked structure of the modified SDF can encapsulate α-glucosidase and α-amylase, inhibiting enzyme activity, thereby delaying the release of glucose, which can achieve the effect of preventing diabetes. Fermented fruit and vegetable SDF can effectively prevent cardiovascular diseases by adsorbing more fats, cholesterol, and bile acids and inhibiting the activity of lipid-degrading enzymes, reducing the absorption of fats by the body. Wang et al. used pomegranate residue as a raw material and fermented it with *Bacillus natto* to obtain the highest SDF content under the optimal fermentation conditions. This substance forms high-viscosity fiber bundles in the intestine, which can absorb a large amount of cholesterol and bile acids, affecting their emulsification and diffusion processes to effectively prevent cardiovascular diseases [83]. Fermented DF can lubricate the intestines, accelerate the excretion of harmful substances, promote better fermentation in the intestines, produce a large amount of short-chain fatty acids, and enhance the abundance of beneficial bacteria such as *Firmicutes* and *Bacteroidetes*, improving the body’s barrier function, strengthening immunity, and preventing intestinal diseases. Zhang’s previous research found that *Lactobacillus plantarum* fermentation can change the monosaccharide composition of asparagus polysaccharide, improve the immune function of SDF, and reduce the occurrence of colorectal cancer, gastric cancer, and other diseases [86].

## 7. Other Processing Methods

### 7.1. Ultra-High-Pressure Technology

The use of ultra-high-pressure (UHP) technology is a non-heat treatment process that uses water as a medium to modify materials at room temperature and below, typically with pressures exceeding 100 MPa. Recently, ultra-high-pressure technology has been attracting more attention due to its advantages in terms of performance, sustainability, and cost [75].

Ultra-high pressure can obviously change the structural characteristics of SDF and its interaction with water, thus leading to changes in its physicochemical properties and functionality [87]. Ultra-high-pressure treatment can alter the monosaccharide composition of fruits and vegetables and increase the total DF and SDF content; particularly, when the pressure is suitable, the content of both reaches the maximum. Huan et al. conducted a study on the structure of DF in pomelo fruits treated with ultra-high pressure. It was found that the SDF content increased significantly from 32.49% ± 0.23% to 41.92% ± 0.32% compared to native DF when the pressure was 400 MPa [88]. According to previous reports, ultra-high pressure and ultrafine grinding have been used to modify pear pomace, and the SDF extraction rate of samples treated with ultra-high pressure can increase from 10.0% to 16.0% [87].

High-pressure processing also enhances the water holding capacity, oil holding capacity, and swelling capacity of SDF, which is related to its porous structure and hydrophobic groups. A previous study found that the swelling performance and cholesterol absorption ability of SDF in citrus fruit were the best under ultra-high pressure. The reason for this may be that high-pressure processing exposes more functional groups by breaking hydrogen bonds within and between SDF molecules, thereby increasing the swelling power. In addition, the WHC, OHC, and puffing degree of apple by-products with cellulose as the main component increased by 1.73 times, 1.18 times, and 3.12 times, respectively, after high-pressure processing, mainly due to an increase in the SDF fraction [89]. Sang et al. reported that pomelo peel IDF can be modified by high-pressure processing, resulting in a 1.36-fold increase in water holding capacity, a 2.02-fold increase in oil holding capacity, and a 1.29-fold increase in the swelling capacity of DF after modification [90].

In addition, HPP improves the binding ability of SDF and bile acid in fruits and vegetables, indicating that it has better in vitro lipid lowering activity. Sang et al. reported that the SDF from Shatian pomelo peel after high-pressure processing has better blood glucose and blood lipid lowering activities, and as a novel ingredient, it has potential application value in lipid-lowering and blood glucose-lowering foods [90]. In conclusion, HPP is an effective method for improving the physical and chemical properties and functionality of DF. Therefore, future research should further explore the modification mechanisms of high-pressure treatment and optimize related parameters to enhance the performance of insoluble dietary fiber (IDF).

### 7.2. High-Pressure Homogenization Technology

High-pressure homogenization (HPH) is a non-thermal processing technique that can be used to prepare emulsions, suspensions, and foams and also modify the properties of SDF. The modification effects of HPH primarily come from the homogenization valve of the positive displacement pump. When the material flows through narrow slits, it experiences significant pressure drops and huge shear forces [91]. Other effects generated during the flow process (such as turbulence, shock waves, and cavitation) will change the molecular structure and break down suspended particles, thus altering the physical and chemical properties of DFs. As we all know, the oil absorption, rheological properties, and viscosity of DF will be affected by this high-pressure homogenization, and its oil and water holding capacity will be improved [92].

**Figure 10:**
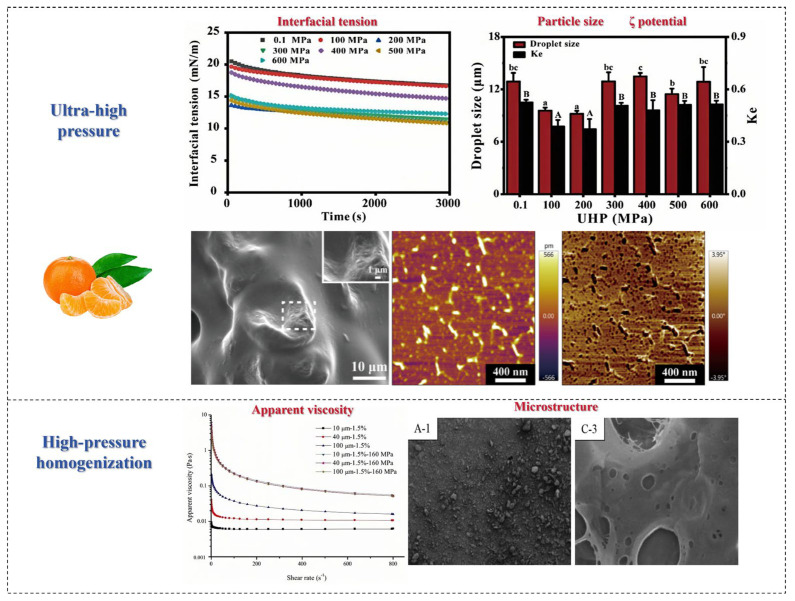
The effects of other processing methods on SDF in fruits and vegetables. Different letters mean significant difference (*p*  <  0.05) [95,96].

HPH can break the intermolecular bonds of DF molecules, refine particles, loosen the structure, significantly reduce the particle size of DF, increase the content of SDF, and improve hydration capacity. However, the strong self-adsorption between ultrafine particles easily leads to particle aggregation during HPH treatment. Adding appropriate emulsifiers can increase the surface potential of DF, thereby enhancing the repulsive effect between particles, which is beneficial for solving the high-pressure aggregation problem. Xie et al. modified purple sweet potato DF with HPH, increasing the content of SDF and total phenols in DF [93]. He et al. studied the effects of HPH on IDF in enoki mushroom, showing that IDF exhibited high water holding capacity, stability, emulsifying properties, and interfacial properties after treatment [94]. Su et al. homogenized citrus DF at different pressures, and the research results showed that the HPH process transformed the spherical citrus fiber particles in the dispersion into a multi-branched flake-like structure, significantly changing the microstructure of citrus fiber in the dispersion. Su et al. used HPH to modify citrus peel SDF, resulting in reduced thermal stability, changes in the microstructure, and improved water holding and oil holding capacities [95]. Figure 10 shows the effects of other processing methods on SDF in fruits and vegetables.

## 8. Comparison of Processing-Induced Structural and Nutritional Variations in Soluble Dietary Fiber in Fruits and Vegetables

### 8.1. Physical Processing Methods

In terms of the structural features of SDF, (1) physical processing may lead to the breakdown of glycosidic bonds in DF monomers, reduce their molecular weight, and thus convert insoluble fibers into soluble fibers, increasing the content of SDF [97]. (2) Physical processing disrupts fiber particles, increases the specific surface area, triggers cell wall rupture, or restructures the fiber molecular structure, further altering the fiber’s solubility and water holding properties [98]. (3) Physical processing may disrupt hydrogen bonding between molecules, leading to a decrease in the crystallinity and polymerization of SDF, causing its structure to become looser and affecting its mechanical strength and thermal stability [54]. (4) Physical processing such as high-pressure treatment, microwave treatment, etc., may alter the internal structure of SDF, such as the arrangement and connection modes of components like cellulose, hemicellulose, and pectin, thus affecting their crystallinity and polymerization degree.

In terms of the nutritional features of SDF, (1) physical processing reduces the molecular weight of SDF, making degraded low-molecular-weight fibers easier to ferment by intestinal microorganisms, producing short-chain fatty acids. These products can stimulate the secretion of intestinal hormones, regulate intestinal flora, and lower blood sugar levels. (2) Physically processed SDF exhibits better hydration properties and oil holding capacity, enhancing its gel formation ability. This gel can delay the absorption of nutrients, effectively regulating postprandial blood glucose levels [99]. (3) After physical processing, the surface area of SDF increases, which helps it to come into contact with digestive enzymes, improves the digestion rate, and enhances the benefits to human health. (4) The fermentability of fibers in physical processing is influenced by the composition of monosaccharides, making it easily fermented by beneficial bacteria in the intestines, increasing the richness and diversity of microorganisms. These probiotics can suppress the growth of harmful bacteria through competition exclusion mechanisms while promoting the proliferation of beneficial bacteria, thus restoring and maintaining the balance of intestinal flora. For example, probiotics such as *Bifidobacteria* and *Lactobacilli* can produce antibacterial substances that inhibit the growth of pathogens while promoting the growth of beneficial bacteria in the intestines, enhancing intestinal barrier function, and improving intestinal immune and infection resistance [100].

### 8.2. Physicochemical Processing Methods

In terms of structural features, (1) Changes in molecular structure: Processing may cause changes in the molecular structure of SDF, such as changes in terms of cellulose hydrolysis and hemicellulose degradation, which can affect its crystallinity, polymerization degree, etc. [101]. (2) Changes in surface properties: During processing, the surface properties of SDF may undergo changes, such as those in surface roughness, hydrophilicity, etc., thereby affecting its contact and interaction with digestive enzymes [102]. (3) Changes in particle size distribution: During processing, the particle size distribution of SDF may change, affecting its digestibility, expansibility, water holding capacity, etc.

In terms of nutritional features, (1) The processing process alters the expandability and digestive absorption characteristics of SDF, forming a gel-like substance in the intestines, which helps to increase digestibility, increase fecal volume, and soften stool. (2) The processing of materialization can make fibers more easily fermented by intestinal microorganisms, increase the production of beneficial short-chain fatty acids, and promote intestinal health. (3) Altering the molecular structure and introducing chemical groups in processing can enhance the fiber’s viscosity and gel-forming ability. High-viscosity fibers can increase intestinal viscosity, delay gastric emptying, and reduce the rate of glucose absorption, thereby weakening postprandial glucose and insulin response [31]. Effects of different physico-chemical processing methods on yield, structure, and properties of SDF are shown in Table 2.

### 8.3. Biological Processing Methods

In terms of structural features, (1) The microbial fermentation of microorganisms can produce several enzymes, such as cellulase, glucosidase, and xylanase [102]. Enzymatic hydrolysis can specifically break the molecular chain of fibers, reducing molecular weight and leading to the formation of more oligosaccharides and monosaccharides [110]. (2) Bioprocessing may make the surface of DF rougher or increase the number of micro-pores. An increase in surface roughness can provide more crystal nuclei, promote crystal growth, and enhance crystallinity. In addition, changes in surface structure may restrict the movement of molecular chains, affect their orderly arrangement, and thereby alter crystallinity. (3) During biological processing, specific enzymes can introduce or remove certain chemical groups (such as carboxyl or hydroxyl groups) to alter the chemical properties of fibers.

In terms of nutritional features, (1) Biological processing releases more beneficial substances. Prebiotics promote the growth and activity of beneficial bacteria in the intestines (such as *bifidobacteria* and *lactobacilli*), improve the balance of intestinal microbiota, enhance digestive function, boost immunity, and reduce the risk of colonization and infection by pathogens. (2) Biologically processed small molecule fibers are more easily fermented by intestinal microbiota, producing beneficial short-chain fatty acids. Short-chain fatty acids can provide energy, regulate intestinal pH, promote mineral absorption, and reduce the risk of colon cancer. (3) Bioprocessing may enhance the antioxidant activity of SDF. Neutralizing excess free radicals in the body, thus reducing oxidative stress and inflammatory reactions, helps protect cells and tissues from oxidative damage. This can help reduce the risk of chronic diseases such as cardiovascular disease, diabetes, and certain types of cancer while also supporting immune function and overall health [111].

The nutritional features of SDF are attributed to its physical and chemical properties, such as water solubility, water retention, swelling, and rheological behaviors. Table 2, Table 3 and Table 4 present the changes in SDF, IDF, and DF content in various food substrates under different processing methods.

## 9. Guidance in Terms of Soluble Dietary Fiber Application in Food Industry

Regardless of whether physical processing, physical–chemical processing, or biological processing is used, the natural structure of SDF is disrupted, the release of SDF is promoted, and the yield is improved. In terms of chemical structure, the long chain structure of dietary fiber is disrupted or hydrolyzed in the three processing methods, leading to a decrease in the molecular weight of SDF and a decrease in viscosity [30]. During processing, the complex polysaccharides of SDF (such as gums) are broken down, converting the originally complex sugar chains into more soluble small molecule sugars. The common result of this is the breakage of polysaccharide chains, resulting in the formation of oligosaccharides or monosaccharides (glucose, xylose, mannose, and arabinose). In the three processing methods, the destruction of the molecular structure of SDF results in the exposure of more hydrophilic functional groups such as hydroxy and carboxy groups, enhancing its solubility in water [2]. In terms of microstructure, the three processing methods can loosen the microstructure of SDF through different mechanisms, exposing more pores and surface area, thus improving its solubility.

Physical processing mainly destroys the cellulose and pectin structures in the cell walls of fruits and vegetables through thermal degradation, releasing soluble components and increasing the content of SDF. Since physical processing mainly changes the structure of SDF through heating without significantly destroying its chemical bonds or molecular structure, the degradation of SDF is minimal, and the increase in SDF content is relatively low. In terms of molecular structure, processing results in the breakage of some macromolecules, which reduces the molecular weight and viscosity of SDF. However, due to the lack of chemical reactions or enzyme participation, the degradation degree is relatively low, resulting in a small decrease in molecular weight and viscosity. In terms of microstructural changes, physical processing mainly alters the surface structure or slightly damages the original form of SDF through thermal effects [121]. Compared to physical and chemical processing, physical processing cannot effectively destroy the cell wall or cause the large-scale breakage of the fiber’s molecular chains. Therefore, the degree of microstructural changes is not significant. Additionally, physical processing (especially high-temperature cooking or drying) disrupts the molecular chain structure, changes hydration and aggregation states, and reduces the hydration ability of SDF [122]. This leads to a more pronounced decrease in water holding capacity and a lower emulsification stability of SDF.

Physical and chemical processing mainly destroys the cell walls and molecular structures of SDF through methods such as expansion and steam explosion, promoting the release of SDF and increasing its content by 2–5 times. Structurally, the combined effects of high temperature, high pressure, and mechanical force in physical and chemical processing lead to a greater extent of molecular chain breakage, resulting in a significant decrease in molecular weight, increased solubility, and a significant decrease in viscosity [97]. At the same time, the destruction of the highly molecular structure of SDF increases its dispersibility and emulsification ability in the water and oil phases, especially in extrusion expansion, which promotes better interaction between the DF surface and fats, thereby enhancing its emulsifying properties [123]. Microscopically, physical and chemical processing leads to the significant rupture of the cell walls of SDF and loose fiber structures, and the microstructure of SDF usually appears fragmented or expanded, which has more significant effects compared to physical processing. In addition, physical and chemical processing methods can effectively improve the functionality of SDF in a short period of time, making them suitable for rapid processing.

The fermentation process in biological processing mainly changes the composition and structure of SDF through the metabolic action of microorganisms and enhances the bioactivity of SDF by producing short-chain fatty acids. The biological processing of other metabolites is mainly achieved through enzymatic hydrolysis or microbial fermentation, selectively degrading certain components in SDF, especially by the action of specific enzymes to selectively break certain sugar chains, releasing more SDF [124]. Additionally, specific enzyme degradation can break down SDF into smaller sugar molecules, resulting in lower molecular weight, reduced viscosity, and improved solubility, making it easier for the human body to digest and absorb. This processing method is milder and more selective, allowing for a targeted reduction in molecular weight. Furthermore, biological processing (such as enzymatic hydrolysis) can selectively degrade complex sugars in SDF, releasing more monosaccharides and oligosaccharides [125]. Therefore, the monosaccharide composition of SDF usually changes after biological processing, producing more monosaccharides and hydrolysis products. Physical and chemical processing can cause the partial breaking of sugar chains, producing a certain amount of monosaccharides or oligosaccharides, but the effect is weaker compared to biological processing. Compared to physical and physical–chemical processing, biological processing can provide additional health benefits to SDF. After fermentation via probiotics, SDF can be efficiently utilized by gut probiotics (such as *lactobacillus* and *bifidobacterium*). By interacting with the intestinal immune system, it can modulate the activity of intestinal immune cells (such as macrophages, T cells, etc.), enhance immunity, and alleviate chronic inflammatory reactions [112].

Specifically, in terms of practical application scenarios, there is the following guiding significance on the application of SDF after different processing process:

Physical processing is suitable for scenarios that require simple, quick, and low-cost processing, and it has especially significant advantages in large-scale production. It is commonly used to improve the texture and quality of SDF, such as in low-fat sausages and vegetarian meat products. Physical processing can promote the binding of SDF with water to form a soft, stable, and firm gel, which can replace some fats and enhance texture [126].

Physical chemistry processing is suitable for scenarios that require significant changes in the functionality of SDF, such as increasing solubility, SDF content, or emulsification, and it is suitable for application in beverage products. For example, in beverages containing fruit and vegetable purees or concentrates, the enhanced emulsification of SDF can improve the stability of the beverage, prevent the precipitation of solid particles in the drink, and improve appearance quality. In dairy alternative beverages such as almond milk and coconut milk, physical chemistry processing can also improve their stability and prevent stratification [127].

Biological processing is suitable for healthy foods and functional foods with high requirements for SDF functionality, especially in increasing SDF content and enhancing prebiotic function. For example, in low-sugar, low-calorie foods, biological processing can convert SDF into prebiotics, producing short-chain fatty acids (such as acetic acid and butyric acid) that can effectively regulate intestinal pH, help improve intestinal function, slow down sugar absorption, and assist in blood sugar control and obesity management [128]. By selecting appropriate processing methods based on specific application scenarios, the functional potential of SDF can be maximized to meet the needs of different consumers and the market.

## 10. Conclusions and Future Prospects

SDF has captured extensive attention due to its functions and physiological benefits. Food processing techniques can alter the structural and nutritional variations in SDF, boosting its bioactivity and offering substantial applications in the food and nutrition sectors. This article systematically reviews the impacts of various processing technologies on SDF’s structure and functional attributes based on existing studies. To gain a deeper understanding of the health impacts of SDF in fruits and vegetables, future research should conduct the following: (1) study the structure–activity relationship of SDF to aid in the targeted modification of dietary fiber and the rational application of food processing technologies to obtain the desired products; (2) determine the optimal beneficial dosage of SDF in diets is essential, which benefits to establish its appropriate intake levels as a next-generation prebiotic.; (3) investigate the regulatory effects of soluble dietary fiber on the composition of the gut microbiota and its mechanisms related to diseases and thus develop dietary fiber intervention strategies for disease management based on these findings.

## Figures and Tables

**Table 2:** Effects of different physico-chemical processing methods on yield, structure, and properties of SDF.

Sources	Method	Technological Treatment	Structural Characteristic	Hydration Characteristic	Biological Activity	References
Enoki mushroom	HPH	0, 10, 30, and 50 cycles at 700 bar	Molecular weight ↓; particle size ↓; void structure ↑; rhamnose, xylose, galactose, and galacturonic acid content ↑	Swelling capacity ↑	Glucose adsorption capacity ↑, cholesterol adsorption capacity ↑	[103]
Bamboo shoot shell	DHPM	100 MPa for 5 passes	Particle size ↓, void structure ↑, crystallinity ↑, thermal stability ↑	Water holding capacity ↑, oil holding capacity ↑	Glucose adsorption capacity ↑, cholesterol adsorption capacity ↑, nitrite adsorption capacity ↑	[100]
Garlic straw	Ultrasound	45 °C, 535 W, 41 min	Molecular weight ↓, particle size ↓, thermal stability ↑	Water retention capacity ↑, oil retention capacity ↑, swelling capacity ↑	Glucose adsorption capacity ↑	[59]
Carrot pomace	UC	Room temperature, standard particle size 40.05 μm	Void structure ↑	Swelling capacity ↑, water retention capacity ↓, oil retention capacity ↓	Cation exchange capacity ↑, glucose adsorption capacity ↑, DPPH radical scavenging capacity ↑, cholesterol adsorption capacity ↑, antioxidant abilities ↑	[104]
Nodes of lotus root	Extrusion	Screw speed 12.56 rad/s, 100 °C	Mannose and xylose content ↑, cellulose and hemicellulose content ↓	Water solubility ↑, water holding capacity ↑, emulsifying activity ↑	-	[105]
Sugarcane bagasse	Extrusion	Constant at 60 °C and 90 °C; 38 rpm	SDF ↑, IDF ↓	Water holding capacity ↑, swelling capacity ↓	-	[106]
Grapefruit peel	Microwave	500 W, 80 °C for 40 min	Molecular weight ↑, crystallinity ↑, thermal stability ↑	Water holding capacity ↑, oil holding capacity ↑	Glucose adsorption capacity ↑, cholesterol adsorption capacity ↑, nitrite adsorption capacity ↑	[107]
Sea buckthorn pomace	Ultrasonic-assisted extraction	70 °C, 105 W, 50 min	SDF ↑	Swelling capacity ↑, water holding capacity ↑, oil holding capacity ↑	-	[108]
*Ipomoea batatas* Lam. residues	Twin-screw extrusion	Screw speed 180 rpm, feed rate 17 Hz, feed moisture 40%, and extrusion temperature 150 °C	SDF ↑, molecular weight ↓, particle size ↓, thermal stability ↑	Swelling capacity ↑, water retention capacity ↑, oil retention capacity ↑	Cholesterol adsorption capacity ↑, glucose adsorption capacity ↑, sodium cholate adsorption capacity ↑	[102]
Coconut residue	Grinding	(1.127–550 μm)	Particle size ↓, void structure ↑	Swelling capacity ↑, water holding capacity ↑, oil holding capacity ↑	Cholesterol adsorption capacity ↓	[109]

**Table 3:** Effects of different bioprocessing methods on yield, structure, and properties of SDF.

Sources	Method	Technological Treatment	Structural Characteristic	Hydration Characteristic	Biological Activity	References
Ginger residue	Cellulase	0.3% cellulase for 60 min at 40 °C	Void structure ↑, IDF ↑	Swelling capacity ↑, water retention capacity ↑, oil binding capacity ↑	Cation exchange capacity ↑, soluble capacity for binding cholesterol↑, soluble nitrite binding capacity ↑	[112]
Potato pulp	Xylanase	pH 5.0, 50 °C, 120 min	SDF ↑,	-	Sodium cholate adsorption capacity ↑, hydroxyl radical scavenging activity ↑	[113]
Bamboo shoot residue	*Inonotus obliquus* fermentation	2 mL of seed culture, for 7 days at 26 °C	SDF ↑	Water holding capacity ↑, oil holding capacity ↑	In vitro cholesterol ↑, sodium cholate adsorption capacity ↑, nitrite adsorption ↑	[114]
Coconut	Cellulase	pH 5.0, 50 °C for 1 h	SDF ↑, cellulase hydrolysis enhanced soluble carbohydrate content↑	Swelling capacity ↑, water holding capacity ↑	α-amino acid interaction ratio ↑, glycemic dietary reference intake ↑, cation exchange capacity ↑	[115]

**Table 4:** Effects of different combined processing methods on yield, structure, and properties of SDF.

Sources	Method	Technological Treatment	Structural Characteristic	Hydration Characteristic	Biological Activity	References
Bamboo shoot	Extrusion and cellulase	60-mesh screen, 20 U/g for 240 min at pH 4.5 and 50 °C	Molecular weight ↑, crystallinity ↑, thermal stability ↑	Water holding capacity ↑, oil holding capacity ↑	Nitrite adsorption capacity ↑, glucose adsorption capacity ↑, cholesterol adsorption capacity ↑	[107]
Rose pomace	Ultrasound, cellulase, and xylanase	150 W for 30 min, hydrolyze 2 h	SDF ↑	Swelling capacity ↑, oil holding capacity ↑	Cholesterol adsorption capacity ↑, cation exchange capacity ↑, glucose adsorption capacity ↓	[116]
Orange peel	Steam explosion and dilute acid	0.8 MPa for 7 min, combined with 0.8% dilute acid	SDF ↑, molecular weight ↓	Water solubility ↑; water holding capacity ↑; oil holding capacity ↑; swelling capacity ↑; emulsifying activity, emulsion stability, and foam stability ↑	Cation exchange capacity ↑	[117]
Papaya peel	Ultrasound and alkaline	170 W, 30 min, 50 °C combined with 1.0% NaOH concentration	SDF ↑, crystallinity ↓, total amino acid content ↓, essential amino acid content ↑, thermal stability ↑	Water holding capacity ↑, oil holding capacity ↑, swelling capacity ↑	-	[118]
*Hovenia dulcis* pomace	Cellulase, xylanase, and UC	Ball mill at 50 r/min for 6 h, then cellulose (10,000 U/mg) and xylanase (100,000 U/mg) incubated at pH 5.0, 50 °C for 120 min	SDF ↑, particle size ↓, zeta potential ↓, disrupted the intermolecular hydrogen bonds and crystalline structure	Water solubility index ↑, apparent viscosity ↑	α-amino acid interaction ratio ↓, α-glucosidase ↓	[119]
Citrus peel	NaOH and HPH	0.5 M NaOH, pH 9.0, for 50 °C, 1 h, and homogenize at 7000 r/min for 25 °C, 25 min	SDF ↑, void structure ↑, crystallinity ↓	Water holding capacity ↑, swelling capacity ↑	-	[120]

## Data Availability

No new data were created or analyzed in this study. Data sharing is not applicable to this article.
